# Supplementary material for: Simplifying Prediction of Intended Grasp Type: Accelerometry Performs Comparably to Combined EMG-Accelerometry in Individuals With and Without Amputation
Source: Sensors (Basel). 2025 Nov 15;25(22):6984. doi: 10.3390/s25226984 (PMC12656353; doi:10.3390/s25226984)
Supplement: Supplementary file 1 [file sensors-25-06984-s001.zip › sensors-3937757-supplementary.pdf]

sensors

MDPI

Article

Simplifying Intended Grasp Type Prediction in Individuals with and Without Amputation: Accelerometry Performs Comparably to Combined EMG/Accelerometry

Samira Afshari *, Rachel V. Vitali and Deema Totah

Department of Mechanical Engineering, University of Iowa, Iowa City, IA 52242, USA; rachel-vitali@uiowa.edu (R.V.V.); deema-totah@uiowa.edu (D.T.)
* Correspondence: samira-afshari@uiowa.edu

Supplementary Material

The supplementary Figure S1 below provides additional details on feature evaluation results across all 468 predictors used in the study. For each participant group (with and without amputation), and for each sensor modality (EMG only, ACC only, and combined EMG+ACC), we present the mean and standard deviation of feature importance scores, computed using a decision tree model. These importance values are expressed as percentages and averaged across all participants within each group. Each predictor corresponds to a specific feature extracted from a single channel, totaling 12 predictors per EMG feature and 36 predictors per ACC feature (12 channels × 3 axes). In the plots, feature groups are visually distinguished using alternating white and gray background shades. These detailed plots provide a full view of the relative contributions of all features considered in the analysis.

check for updates

Academic Editor: Giovanni Saggio

Received: 2 October 2025
Revised: 7 November 2025
Accepted: 12 November 2025
Published:

Citation: Afshari, S.; Vitali, R.V.; Totah, D. Simplifying Intended Grasp Type Prediction in Individuals with and Without Amputation: Accelerometry Performs Comparably to Combined EMG/Accelerometry. *Sensors* **2025**, *1*, 0. https://doi.org/

Copyright: © 2025 by the authors. Licensee MDPI, Basel, Switzerland. This article is an open access article distributed under the terms and conditions of the Creative Commons Attribution (CC BY) license (https://creativecommons.org/licenses/by/4.0/).

Subjects with amputation **Able-bodied subjects**

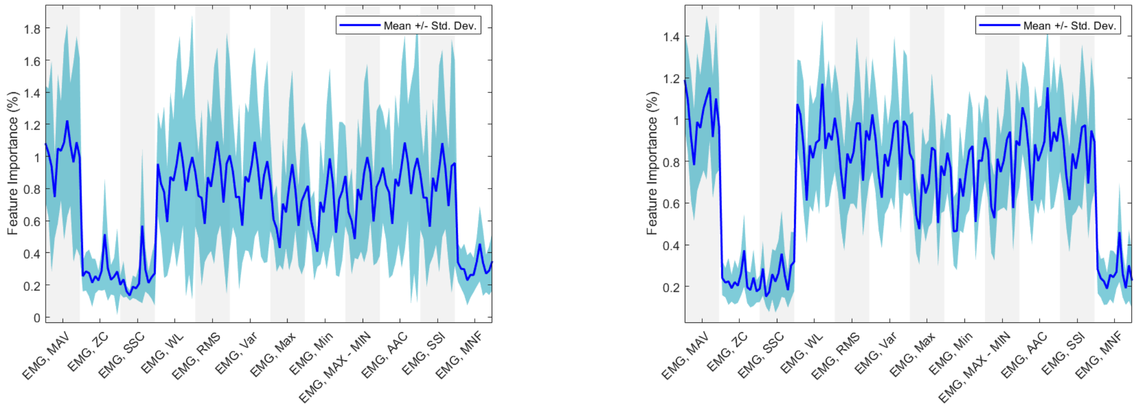

(a) EMG Features

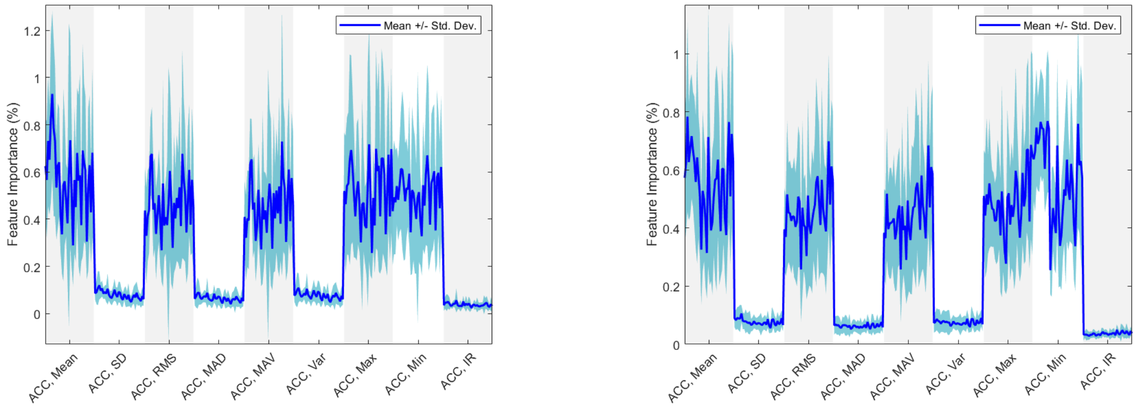

(b) ACC Features

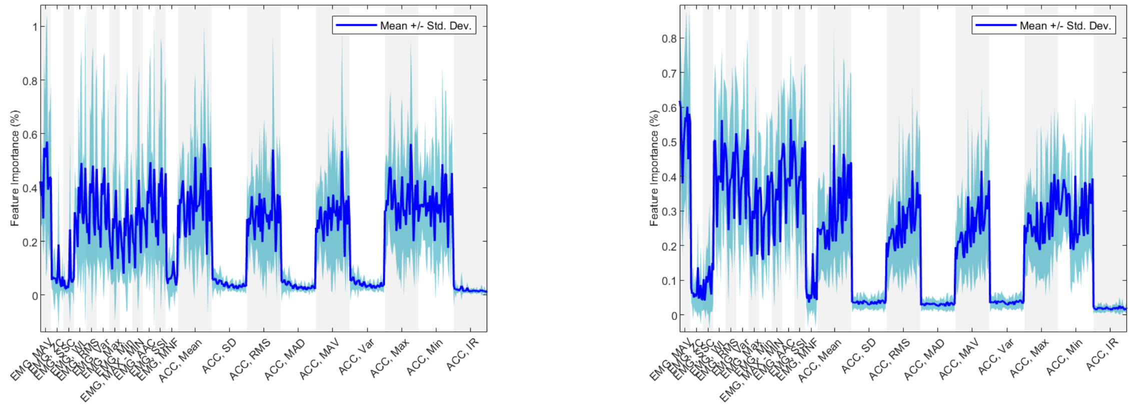

(c) EMG and ACC Features

Figure S1. Feature Importance Scores Across Subjects. Mean and standard deviation values for feature importance percentage across subjects, when features are scored for (a) EMG only data, (b) ACC only data, (c) both EMG and ACC data. All scores are labeled in feature groups of channels. Shades of white and light gray show all the predictors for the corresponding feature group. Three plots on the left column are for subjects with amputation and three plots on the right column are for able-bodied subjects.

Table S1. Subject-specific Classification Accuracies. Subject-specific LDA classification accuracies (%) for amputated (trans-radial) and able-bodied participants across sensing modalities, trained with all features. The table illustrates inter-subject variability within each group.

**Subject**	**EMG**	**ACC**	**EMG+ACC**
**Participants with amputation (AMP)**			
AMP1	54.35	87.60	89.64
AMP2	38.96	84.30	85.48
AMP3	47.29	76.94	81.06
AMP4	46.45	77.57	81.80
AMP5	54.10	86.20	88.63
AMP6	52.82	78.99	82.91
AMP7	50.69	88.29	90.01
AMP8	61.75	89.98	92.64
AMP9	53.83	76.10	82.55
AMP10	57.31	86.61	90.10
AMP11	61.61	88.71	91.66
AMP12	59.70	83.44	87.25
AMP13	65.20	92.22	93.90
**Able-bodied participants (AB)**			
AB1	55.46	83.18	87.55
AB2	56.24	75.67	80.10
AB3	64.89	79.09	87.77
AB4	67.60	85.86	90.50
AB5	56.63	81.55	85.74
AB6	61.98	82.78	86.42
AB7	54.52	82.38	86.28
AB8	70.75	90.49	94.32
AB9	52.41	83.41	86.96
AB10	54.13	80.73	84.63
AB11	68.27	84.64	89.78
AB12	59.72	89.38	92.33
AB13	65.65	87.45	91.20
AB14	66.78	89.03	92.40
AB15	68.59	86.18	90.56
AB16	62.15	85.53	88.54
AB17	63.17	83.00	88.03
AB18	71.52	87.64	92.10
AB19	64.41	88.11	90.76
AB20	61.49	84.16	88.60
AB21	62.15	83.75	88.69
AB22	60.52	84.81	89.98
AB23	62.33	88.76	91.64
AB24	57.70	87.58	90.34
AB25	59.66	86.50	88.88
AB26	64.44	88.49	92.55
AB27	61.01	81.44	87.82
AB28	60.78	87.54	90.75

Confusion matrices (Figure S2) and recall values (Figure S3) from an LDA model trained with all features show class-level recall (mean ± SD %, across subjects). Diagonal elements represent correctly classified grasp types, while off-diagonal values indicate misclassifications. Most errors occurred between functionally or kinematically similar grasps, such as precision disk vs. power sphere. In both participant groups, multimodal fusion (EMG + ACC) improved class separability, particularly reducing confusion among precision and power grasps. ACC-only classification showed greater overlap among grasps involving subtle wrist motion, whereas EMG-only performance was limited by inter-class similarity in muscle activation patterns. These results suggest that most residual errors stem from functional overlap rather than sensor noise. Targeted training or additional sensing focused on finger and thumb motion, as well as a small secondary classifier dedicated to distinguishing these specific pairs, could potentially improve performance.

Subjects with amputation | **Able-bodied subjects**

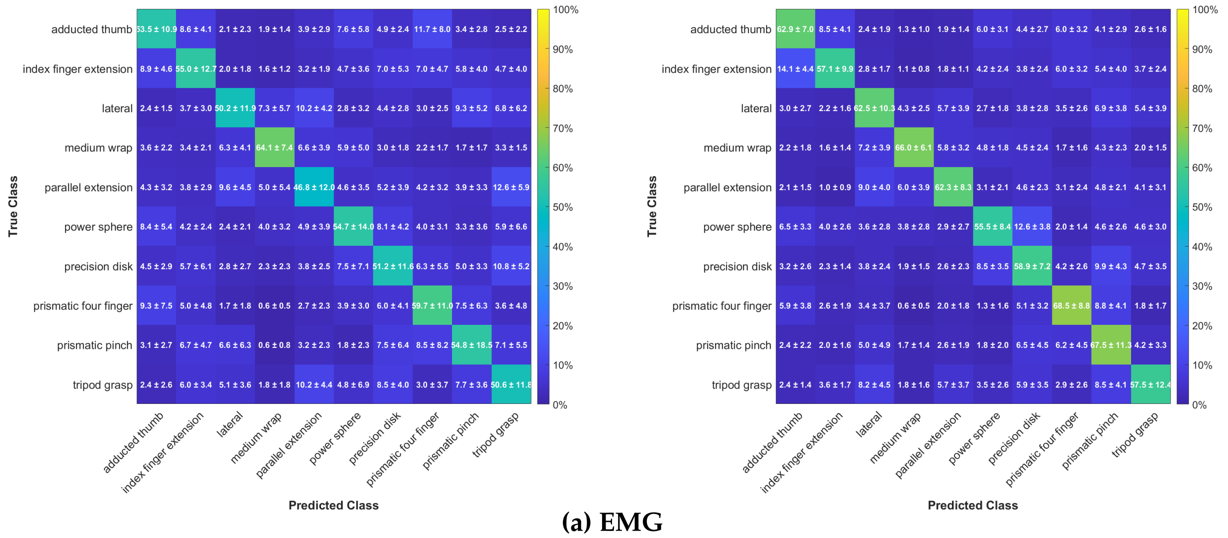

(a) EMG

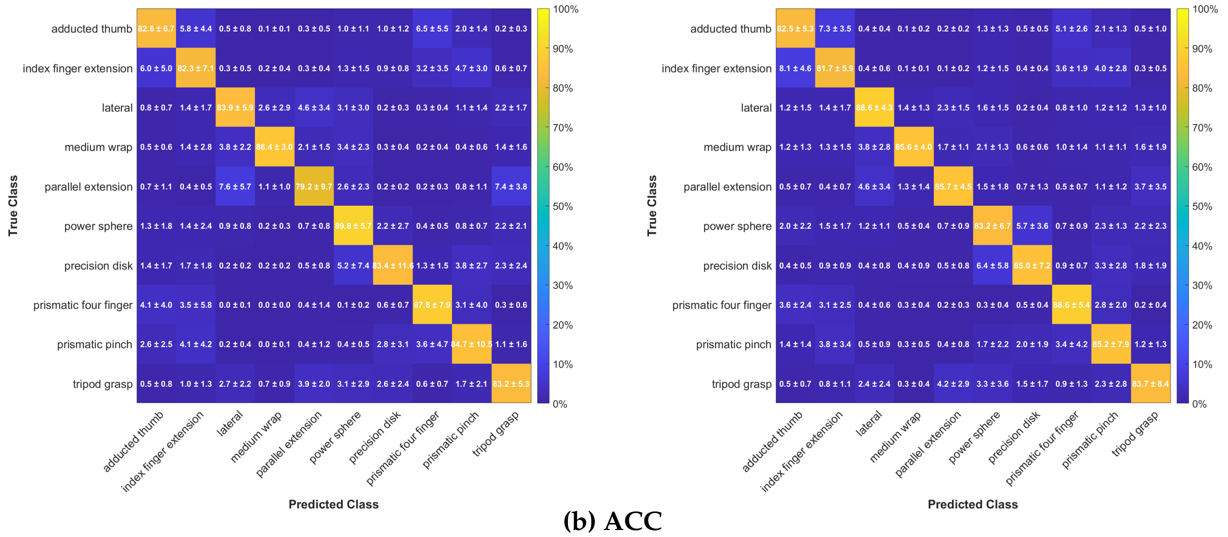

(b) ACC

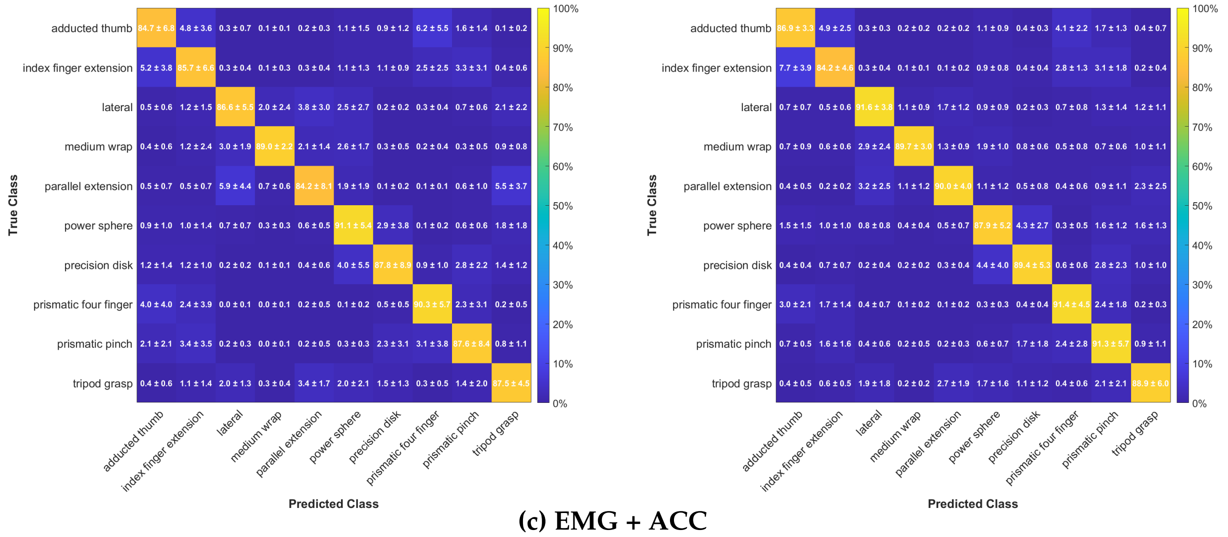

(c) EMG + ACC

Figure S2. LDA Classifier Confusion Matrices. Confusion matrices showing class-level recall (mean± SD %, across subjects) for EMG, ACC, and EMG + ACC modalities separately for participants with and without amputation.

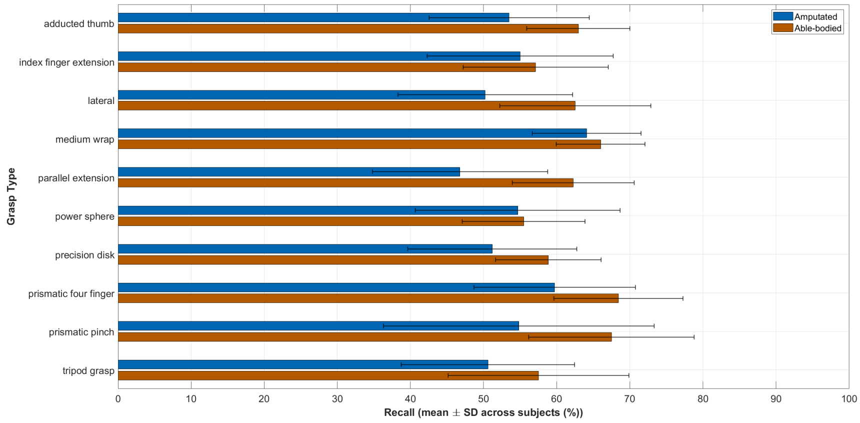

(**a**) EMG

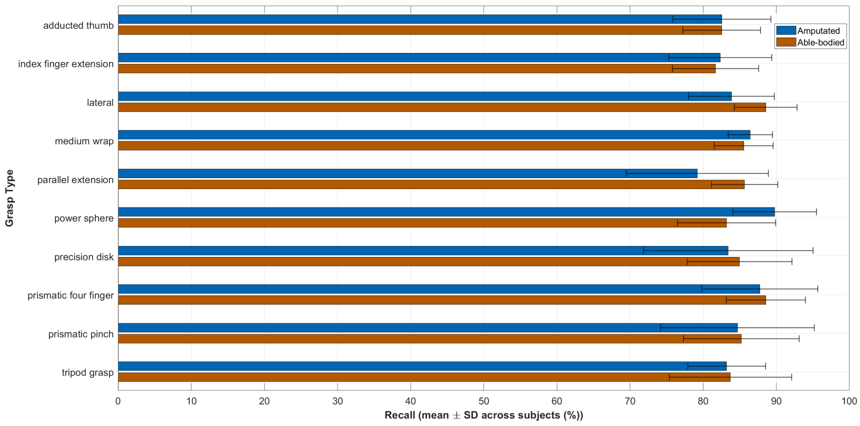

(**b**) ACC

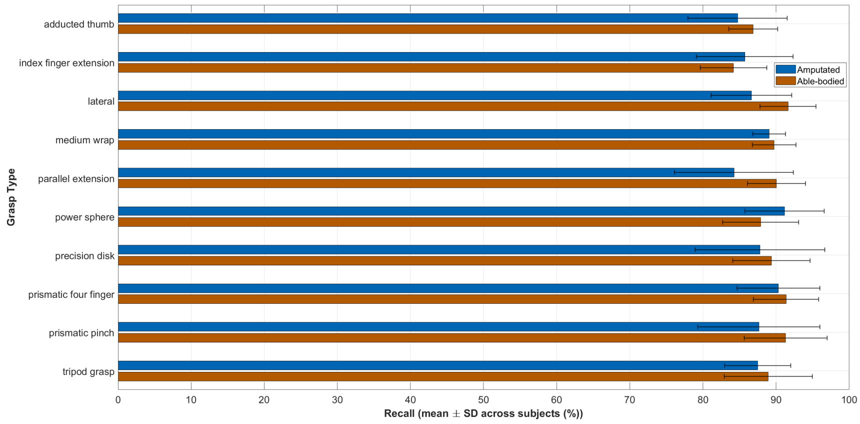

(**c**) EMG + ACC

Figure S3. LDA Classifier Recall Performance. Percentage recall values (mean ± standard deviation across subjects) for each of the ten grasp types compared between two groups of participants when using (a) only EMG, (b) only ACC, and (c) combined EMG + ACC.
